# Exercise cardiovascular magnetic resonance reveals reduced cardiac reserve in pediatric cancer survivors with impaired cardiopulmonary fitness

**DOI:** 10.1186/s12968-020-00658-4

**Published:** 2020-09-07

**Authors:** Stephen Foulkes, Benedict T. Costello, Erin J. Howden, Kristel Janssens, Hayley Dillon, Claudia Toro, Piet Claus, Steve F. Fraser, Robin M. Daly, David A. Elliott, Rachel Conyers, Andre La Gerche

**Affiliations:** 1 Department of Sports Cardiology, Baker Heart and Diabetes Institute, 75 Commercial Rd, Melbourne, VIC 3004 Australia; 2 Institute for Physical Activity and Nutrition, Deakin University, Geelong, VIC Australia; 3 Cardiology Department, St Vincent’s Hospital Melbourne, Melbourne, VIC Australia; 4 Children’s Cancer Centre, The Royal Children’s Hospital, Melbourne, VIC Australia; 5 Department of Cardiovascular Sciences, KU Leuven, Leuven, Belgium; 6 Murdoch Children’s Research Institute, The Royal Children’s Hospital, Melbourne, VIC Australia; 7 Department of Paediatrics, University of Melbourne, Parkville, VIC Australia; 8 Medicine, Dentistry and Health Sciences, University of Melbourne, Parkville, VIC Australia

**Keywords:** Cardiotoxicity, Cardiac function, Cardiopulmonary fitness, Cancer, Exercise CMR

## Abstract

**Background:**

Pediatric cancer survivors are at increased risk of cardiac dysfunction and heart failure. Reduced peak oxygen consumption (peak VO_2_) is associated with impaired cardiac reserve (defined as the increase in cardiac function from rest to peak exercise) and heart failure risk, but it is unclear whether this relationship exists in pediatric cancer survivors. This study sought to investigate the presence of reduced peak VO_2_ in pediatric cancer survivors with increased risk of heart failure, and to assess its relationship with resting cardiac function and cardiac haemodynamics and systolic function during exercise.

**Methods:**

Twenty pediatric cancer survivors (8–24 years; 10 male) treated with anthracycline chemotherapy ± radiation underwent cardiopulmonary exercise testing to quantify peak VO_2_, with a value < 85% of predicted defined as impaired peak VO_2_. Resting cardiac function was assessed using 2- and 3-dimensional echocardiography, with cardiac reserve quantified from resting and peak exercise heart rate, stroke volume index (SVI) and cardiac index (CI) using exercise cardiovascular magnetic resonance (CMR).

**Results:**

Twelve of 20 survivors (60%) had reduced peak VO_2_ (70 ± 16% vs. 97 ± 14% of age and gender predicted). There were no differences in echocardiographic or CMR measurements of resting cardiac function between survivors with normal or impaired peak VO_2_. However, those with reduced peak VO_2_ had diminished cardiac reserve, with a lesser increase in CI and SVI during exercise (Interaction *P* < 0.01 for both), whilst the heart rate response was similar (*P* = 0.71).

**Conclusions:**

Whilst exercise intolerance is common among pediatric cancer survivors, it is poorly explained by resting measures of cardiac function. In contrast, impaired exercise capacity is associated with impaired haemodynamics and systolic functional reserve measured during exercise. Consequently, measures of cardiopulmonary fitness and cardiac reserve may aid in early identification of survivors with heightened risk of long-term heart failure.

## Background

Current 5-year survival rates for pediatric malignancies in developed nations have increased from 58% in 1975–1977 to 83% for those diagnosed between 2005 and 2011 [1]. However, these dramatic improvements in cancer-related survival have accentuated the long-term consequences of cancer treatment, with survivors of pediatric malignancies experiencing increased morbidity and early mortality due to treatment-related chronic health conditions [2–4]. Cardiovascular disease is the most common morbidity experienced by pediatric cancer survivors with 11% of survivors having a diagnosable cardiac condition prior to 40 years of age, of which heart failure is the predominant cause [5]. This has led to a shift in focus, whereby treatment success is determined by the balance between treatment efficacy and the risk of treatment-related toxicities [6]. However, despite international recommendations, less than 28% of survivors are receiving appropriate cardiac screening [7].

The current paradigm for preventing cardiotoxicity relies on the early detection of cardiac dysfunction when it may be more amenable to initiation of heart failure therapy [8]. Clinical decision making is based on serial changes in resting left ventricular (LV) ejection fraction (LVEF) or LV fractional shortening (FS), which is assessed using two-dimensional (2D) echocardiography or radionucleotide ventriculography [8]. The identification of LVEF or LV FS below the lower limit of normal should prompt the initiation of cardioprotective heart failure therapies [6, 8]. For those patients who are undergoing active treatment, this may also result in treatment modification, delay or cessation [6]. However, clinical decision making is complicated by the lack of sensitivity of LVEF and LV FS in explaining treatment-induced functional limitations, patients’ symptoms and longer-term heart failure events [9, 10]. This has resulted in increased interest in alternative surveillance methods for identifying cardiac dysfunction and quantifying heart failure risk [8]. The assessment of myocardial deformation via novel resting echocardiographic or cardiovascular magnetic resonance (CMR) methods are emerging as more sensitive markers of sub-clinical cardiac dysfunction in various cancer groups. However, the degree to which these measures are associated with functional impairment and quantifiable heart failure symptoms such as exercise intolerance is unclear.

Exercise intolerance, defined as reduced cardiopulmonary fitness or peak oxygen consumption (peak VO_2_) measured from a cardiopulmonary exercise test (CPET), has proven clinical utility in quantifying heart failure symptoms and predicting prognosis [11, 12]. Recent advances in cardiac imaging have enabled the measurement of cardiac function augmentation with exercise (termed “cardiac reserve”) to be assessed with greater accuracy and have been shown to be strongly associated with peak oxygen consumption (VO_2_ ) [13]. Thus, both peak VO_2_ and cardiac reserve may provide greater sensitivity for detecting sub-clinical cardiac dysfunction than resting measures [12]. However, associations between exercise intolerance and cardiac function (including cardiac reserve) in pediatric cancer survivors remain inconclusive [14, 15].

Therefore, the aim of this study was to explore the presence of exercise intolerance in survivors of childhood and adolescent malignancy at high risk of cardiac dysfunction, and to explore its relationship with measures of resting cardiac function, and exercise-based measures of cardiac haemodynamics and systolic function.

## Methods

### Participant population and study design

Participants were cancer survivors treated or undergoing active treatment for pediatric haematologic malignancy at the Royal Children’s Hospital, Monash Medical Centre or Alfred Hospital, Melbourne. Participation involved recruitment from attendance at routine outpatient clinics, with participants providing informed written consent prior to participation in the study. This study was approved by the ethics committee of the Melbourne Royal Children’s Hospital (HREC 35102D) and the Alfred Hospital (HREC 00315) and was conducted in accordance with the Declaration of Helsinki guidelines.

Enrollment criteria included: (1) previous treatment with anthracycline chemotherapy with or without craniospinal or chest-targeted radiotherapy, and (2) height > 120 cm (in order to reach pedals for exercise testing). Participants were excluded if they had a previous history of severe symptomatic cardiac disease or had a contraindication to CMR.

### Outcome measures

Comprehensive resting and exercise evaluation was completed at the Baker Heart and Diabetes Institute, Melbourne. Participants were evaluated with i) comprehensive resting transthoracic echocardiogram to assess LVEF, LV FS and global longitudinal strain (GLS), ii) a maximal CPET to evaluate peak VO_2_ as an indication of cardiopulmonary fitness, and iii) resting and exercise CMR (exCMR) to determine resting GLS, and resting and peak exercise LVEF, right ventricular (RV) ejection fraction (RVEF), stroke volume index (SVI), heart rate and cardiac index (CI) as measures of cardiac reserve. Participants were classified by normal peak VO_2_ or impaired peak VO_2_ defined as peak VO_2_ ≥ 85% age-predicted values. Cardiovascular risk factors were assessed at the time of study enrollment. Participants were assessed for hypertension, diabetes mellitus, chronic renal insufficiency and body mass index (BMI). Haemoglobin concentration was obtained from routine clinical blood results conducted within 2 months of the study visit, and participants were also screened for pulmonary comorbidities that could provide an alternative explanation for exertional intolerance.

#### Exercise capacity

CPET was conducted on an electronically braked cycle ergometer (Lode Excalibur, Groningen, The Netherlands) for the measurement of peak VO_2_. Workload was increased using a ramp protocol which began at 10–25 watts (W) and progressively increased at 10–30 W/min until volitional exhaustion. Respiratory gas analysis was measured continuously throughout the test using a calibrated metabolic cart (True One 2400, Parvomedics, Salt Lake City, Utah, USA). Heart rate, rhythm and presence of ischemia was measured continuously by 12-lead electrocardiogram (ECG) (Norav Medical, Wiesbaden, Germany). Peak V̇O_2_ was defined as a 30-s rolling average of the six highest 5-s oxygen uptake values. Participants were classified as having impaired peak VO_2_ if they achieved < 85% of predicted values for children/adolescents [16] or adults [17] in line with American Thoracic Society guidelines for CPET interpretation [18]. The anaerobic threshold was calculated using the V-slope method [19], with the VO_2_ at the anaerobic threshold expressed as an absolute value and as a proportion of each participant’s predicted peak VO_2_.

### Echocardiography

Resting cardiac function was assessed from a comprehensive resting echocardiogram (Vivid E95, General Electric Healthcare, Milwaukee, Wisconsin, USA), with images saved in a digital format for offline analysis (Echopac v13.0.00, General Electric Healthcare). A full-volume three-dimensional data set was acquired. LV end-diastolic and end-systolic volumes were measured according to standard recommendations [20]. Two-dimensional GLS was quantified from three apical views at a temporal resolution of 60–90 frame/s. The average negative value on the strain curve was reported as GLS. Doppler measures of diastolic function were acquired and analyzed per guideline recommendations [21].

### CMR imaging

CMR imaging was performed with a 3 T CMR system (MAGNETOM Prisma, Siemens Healthineers, Erlangen, Germany) with a 5-element phased array coil. Ungated real-time balanced steady state free-precision cine imaging was performed with a parallel imaging acceleration factor of 3 and subsequent GRAPPA reconstruction without cardiac or respiratory gating. Forty (during exercise) or 100 (at rest) consecutive frames were acquired with a temporal resolution of 39 ± 3 msec in a short- and long-axis plane. Typical imaging parameters were field of view = 360 mm, 128 × 128 matrix, voxel size = 2.8 × 2.8 × 8 mm, slice gap = 0 mm, echo time = 1.18 ms, echo spacing = 2.6 ms, flip angle = 48°-68°, bandwidth = 1260 Hz/Px .

#### Resting global longitudinal strain

GLS was calculated using feature tracking on the cine images (CVI42, Circle Cardiovascular Imaging Inc. Calgary, Alberta, Canada). The myocardium was defined according to American Heart Association segments by placing a marker across the mitral valve annulus and from the annulus to the apex on long axis images, and by marking endocardial and epicardial borders in the short axis volumetric stack and three apical cine images (4 chamber, 2 chamber, 3 chamber). Markers were placed at both RV insertion points on the short axis images. The feature-tracking algorithm within the CVI42 software calculated GLS.

#### Exercise CMR imaging

The biventricular response to exercise was measured using a real-time CMR protocol that has been described in detail previously and validated against invasive measures [13]. Images were obtained at rest, and whilst cycling using a CMR compatible ergometer (MR Ergometer Pedal, Lode, Groningen, Netherlands) at an intensity equal to 60% of maximal power output obtained during the upright incremental cycle exercise test (demonstrated in an additional movie file - Additional file 1) which we have previously shown approximates near maximal exercise in a supine position [13]. Exercise was maintained for up to 3 min; 30 s to achieve a physiological steady-state and approximately 1 to 2.5 min for image acquisition.


**Additional file 1** *Example of a pediatric cancer survivor with good and poor biventricular haemodynamic and contractile reserve assessed by exercise cardiac magnetic resonance imaging.* The survivor with poor cardiac reserve showed little augmentation in stroke volume (Rest: 42 mL/m^2^ vs Peak: 44 mL/m^2^), LVEF (Rest: 53% vs Peak: 57%) or RVEF (Rest: 52% vs Peak: 56%), and had a peak VO_2_ 73% of predicted. In contrast, the survivor with good cardiac reserve showed significant augmentation of stroke volume (Rest: 42 mL/m^2^ vs Peak: 50 mL/m^2^), LVEF (Rest: 55% vs Peak: 66%) and RVEF (Rest: 54% vs Peak: 66%), and had a peak VO_2_ that was 99% of predicted.

Images were analyzed on a software program developed in-house (RightVol – Right Volume Leuven, Leuven, Belgium) with retrospective respiratory and ECG gating to ensure endocardial contouring could be performed at the same point in the respiratory cycle. LV and RV endocardial contours were then manually traced on the short axis image with simultaneous reference to the horizontal long axis plane, thereby enabling constant referencing of the atrioventricular valve plane. Ventricular volumes were calculated by a summation of disks, with trabeculations and papillary muscle considered part of the ventricular blood pools. SVI was measured as the difference between end-diastolic volume and end-systolic volumes indexed to body surface area, while CI was calculated as (RV SV + LV SV/2) × heart rate (HR) indexed to body surface area. Using this technique, our group has demonstrated excellent inter-observer (R = 0.98 and R = 0.97 for LV and RV SV, respectively) and inter-study reproducibility (R = 0.98 for cardiac output) [13]. Peripheral muscle arterio-venous oxygen difference (a-vO_2_ diff) was calculated indirectly according to the Fick principle [22], using peak V̇O_2_ from the CPET and peak cardiac output measured by exercise CMR.

#### Statistical analysis

Data analyses were conducted using SPSS statistical software (version 24, Statistical Package for the Social Sciences, International Business Machines, Inc., Armonk, New York, USA). The distribution of continuous variables was tested using a Kolmogorov-Smirnov test. Categorical variables are expressed as n (%), whilst continuous variables are expressed as mean **±** standard deviation or median (interquartile range) as appropriate. Differences in participant characteristics were compared by independent t-tests or Mann-Whitney U tests for continuous variables, and chi-square or fisher’s exact tests for categorical variables. Differences in the exCMR cardiac response to exercise was assessed using repeated measures ANOVA (with an interaction term for group x exercise response), with post-hoc analysis conducted using Bonferroni correction. Pearson correlations were used to test for associations between peak VO_2_ and measures of cardiac function and peripheral muscle oxygen extraction. A 2-sided *P* value of < 0.05 was considered statistically significant.

## Results

### Participant characteristics

A total of 20 patients participated in the study, and the summary of participant demographics and characteristics are summarized in Table 1. The median age of participants was 17 years (range 8–24), of which 10 were male (50%). The most common tumor type was acute lymphoblastic leukemia (*n* = 10, 50%). The median anthracycline dose was 175 mg/m^2^ with most participants (*n* = 16) receiving between 150 and 350 mg/m^2^ and two participants exceeding 450 mg/m^2^. Six participants had radiation together with chemotherapy, of which two received total body radiation, two received cranio-spinal radiation, and two received field radiotherapy to the chest. Two survivors had previously undergone allogeneic stem cell transplantation two years previously. There were no pulmonary comorbidities in the study cohort, however seven participants (35%) had at least one cardiovascular disease risk factor. Both groups had a similar BMI (Normal: 21.7 ± 4.1 kg/m^2^ vs Impaired: 20.4 ± 4.0 kg/m^2^, *P* = 0.45) and haemoglobin concentration (Normal: 135 g/L [104, 184] vs Impaired: 128 g/L [93, 144], *P* = 0.18). However, the impaired fitness group tended to have a higher proportion of females (67% vs 25%, *P* = 0.085) and tended to have a shorter time from initial diagnosis (median 2.6 vs 5.1 years, *P* = 0.076). There were no other significant differences in demographic, diagnosis or treatment characteristics between groups.

**Table 1:** Demographic and treatment characteristics (*n* = 20)

Current age (years)	17 (8–24)
Age at diagnosis (years)	13 (6–21)
Time since diagnosis (years)	4.4 (0.5–15.3)
Peak VO_2_ (L/min)	1.9 ± 0.9
% Predicted peak VO_2_ (%)	81 ± 20
Haemoglobin, g/L	129 (93, 184)
**Sex**	
Male	10 (50%)
Female	10 (50%)
**Diagnosis**	
Acute lymphoblastic leukaemia	12 (60%)
Acute myeloid leukaemia	5 (25%)
Hodgkin rumour	2 (10%)
Wilms rumour	1 (5%)
**Treatment Details**	
Anthracycline, cumulative dose exposure (mg/m^2^)	175 (89–670)
Chest-targeted radiation	2 (10%)
Craniospinal radiation	2 (10%)
Total body radiation	2 (10%)
Allogeneic SCT	2 (10%)
**Cardiovascular risk factors**	
Hypertension	1 (5%)
Diabetes	2 (10%)
Dyslipidaemia	3 (15%)
Chronic renal insufficiency	1 (5%)
BMI > 25 kg/m^2^	3 (15%)

Values are mean ± SD, median (range) or n (%). Abbreviations: *SCT* Stem cell transplantation, *BMI* Body mass index

### Exercise capacity

Differences in exercise capacity and CPET parameters for those with normal (*n* = 8) and impaired peak VO_2_ (*n* = 12) are summarized in Table 2. All subjects reached > 85% predicted maximal heart rate, with all but one subject obtaining a peak respiratory exchange ratio > 1.1 indicative of exercise to maximal or near-maximal intensity. Survivors with impaired fitness achieved a peak VO_2_ that was on average 30% below predicted values, compared to 3% below predicted for survivors with normal peak VO_2_. Survivors with impaired fitness achieved a significantly lower absolute (*P* = 0.018) and body-weight indexed peak VO_2_ (*P* = 0.003) to those with normal fitness, whilst also tending to achieve a lower peak workload (*P* = 0.089). In contrast, the heart rate and ventilatory response was similar (*P* > 0.05 for all variables) with the exception of a tendency for a higher peak respiratory exchange ratio in the impaired fitness group (*P* = 0.086). Survivors with impaired fitness also had significantly lower VO_2_ at the AT (*P* = 0.025), which on average, occurred at 50% of their predicted peak VO_2_, compared to 66% of predicted peak VO_2_ in the normal fitness group (*P* = 0.02).

**Table 2:** Exercise capacity and cardiopulmonary parameters

	Normal Fitness	Impaired Fitness	P
**Cardiopulmonary Exercise Testing**			
*% Predicted HR*_*max*_ *(%)*	99 ± 7	98 ± 5	0.65
*Respiratory exchange ratio*	1.20 ± 0.07	1.33 ± 0.20	0.086
*Peak Workload (watts)*	234 ± 89	156 ± 85	0.089
*Exercise Time (mins)*	9.0 (6.0, 12.0)	8.5 (5.3–11.8)	0.22
*Peak VO*_*2*_ *(L/min)*	2.5 ± 1.1	1.5 ± 0.7	0.018
*Peak VO*_*2*_ *(mL/kg/min)*	40.8 ± 10.0	27.3 ± 7.4	0.003
*% Predicted Peak VO*_*2*_ *(%)*	97 ± 14	70 ± 16	0.001
*VO*_*2*_ *at AT (L/min)*	1.7 ± 0.7	1.1 ± 0.4	0.025
*% Predicted Peak VO*_*2*_ *at AT (%)*	66 ± 12	50 ± 14	0.02

Values are mean ± SD or median (interquartile range). Abbreviations: *AT* Anaerobic threshold, *HR* Heart rate, *RER* Respiratory exchange ratio

### Resting cardiac function

**Fig. 1:**
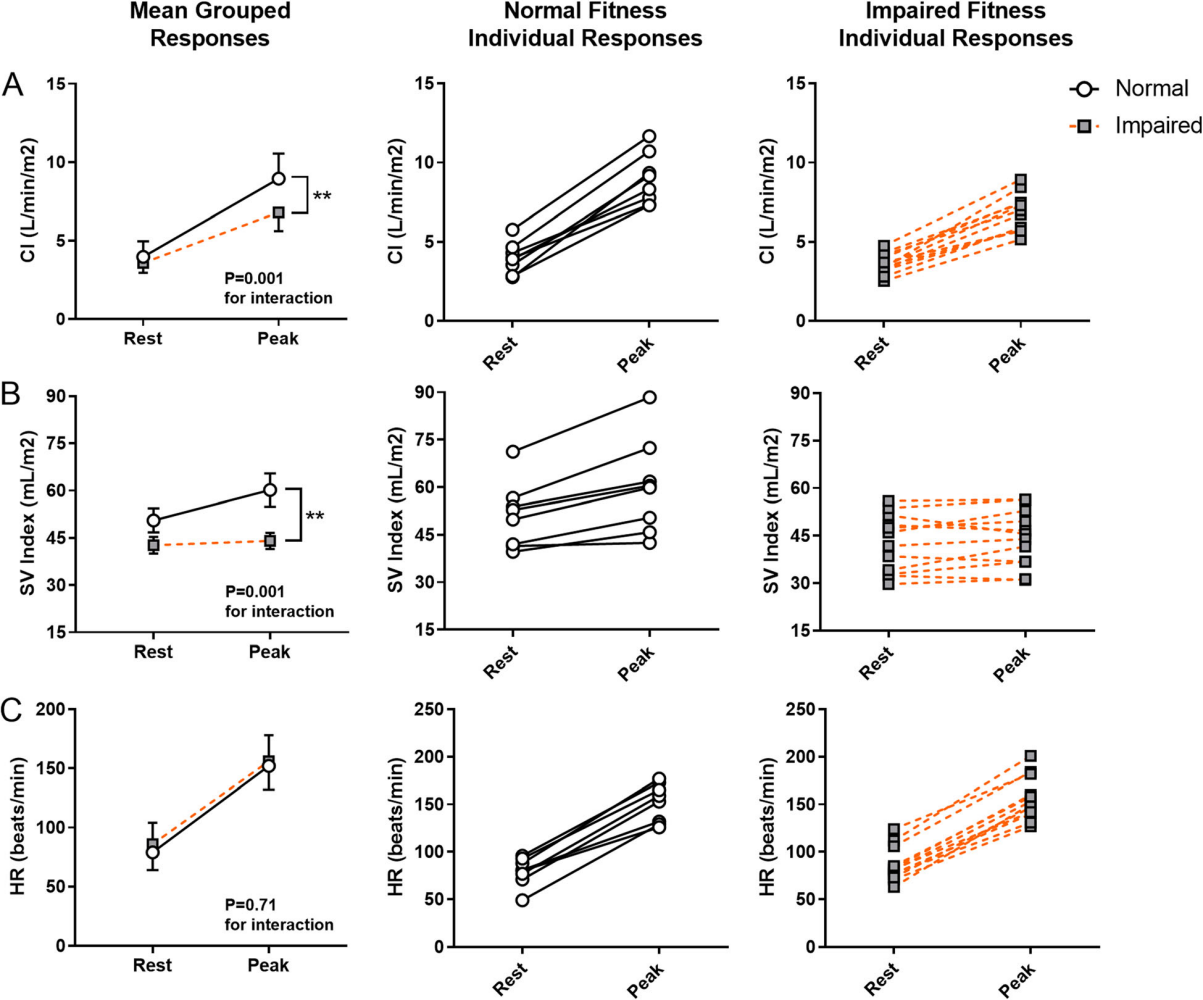
Individual cardiac hemodynamic response to exercise in pediatric cancer survivors with normal or impaired cardiopulmonary fitness. Survivors with impaired fitness had a blunted increase in cardiac index (CI) compared to survivors with normal fitness (Fig. 1a). Stroke volume index (SVI) was lower in the impaired fitness group, with minimal augmentation from rest to peak exercise (Fig. 1b), whilst the heart rate response was similar (Fig. 1c)

Results for resting echocardiographic and CMR measures of cardiac function for both groups are summarized in Table 3. Echocardiographic and CMR-derived GLS could not be obtained in one participant due to sub-optimal image quality. Despite marked differences in exercise capacity, there were no significant differences in any measures obtained from resting echocardiography, with both groups showing similar resting GLS (Supplementary Fig. 1). Three participants (38%) with normal fitness and five participants (42%) with impaired fitness were classified with reduced LVEF (≤53%), whilst two with normal fitness (25%) and three with impaired fitness (25%) were classified with reduced LV FS (< 28%). There were no differences in the proportion of each group with impaired resting LVEF or FS (*P* > 0.05 for both measures). Resting CMR-derived LVEF was similar between groups, whilst RVEF was significantly higher (*P* = 0.006) and SVI tended to be lower (*P* = 0.09) in the impaired fitness group. In contrast, there were no differences in resting CI (Fig. 1), nor CMR-derived GLS (Table 3; also see Additional file 1, Supplementary Fig. 1 which illustrates the individual values for GLS for both groups).

**Table 3:** Resting and peak cardiac function

	Normal Fitness	Impaired Fitness	P
***Resting Echocardiography***			
LVEF, %	55.1 ± 3.6	55.3 ± 8.6	0.97
LV FS, %	32.9 ± 5.4	30.5 ± 5.0	0.32
GLS, %	−18.6 ± 2.4	− 17.8 ± 2.1 ^a^	0.46
E/A	1.87 (1.09, 2.65)	1.79 (0.77, 2.81)	0.79
E/e’	6.0 (4.4, 7.6)	6.8 (5.1, 8.5)	0.26
DT, cm/s	183 ± 23	176 ± 64	0.64
***Resting CMR***			
GLS, %	−15.3 ± 2.3	−14.9 ± 2.0 ^b^	0.69
LVEF, %	53.8 ± 3.8	52.6 ± 6.6	0.66
RVEF, %	49.4 ± 4.0	55.6 ± 4.8	0.006
SVI, mL/m^2^	51 ± 11	43 ± 9	0.09
CI, L/min/m^2^	4.0 ± 1.0	3.6 ± 0.6	0.29
***Exercise CMR***			
LVEF_peak_, %	65.0 ± 3.2	60.0 ± 7.8	0.07
RVEF_peak_, %	62.8 ± 5.1	63.9 ± 6.3	0.67
SVI_peak_, mL/m^2^	60 ± 15	44 ± 9	0.007
CI_peak_, L/min/m^2^	9.0 ± 1.6	6.8 ± 1.2	0.003
a-vO_2_ diff^c^	16.3 ± 3.3	12.9 ± 2.5	0.016

Values are means ±SD or median (interquartile range). *LV* Left ventricular, *LVEF* Left ventricular ejection fraction, *FS* Fractional shortening, *GLS* Global longitudinal strain, *E/A* Ratio of peak early-to-late mitral inflow velocities, *E/e’* Ratio of peak mitral inflow to peak early mitral annular velocity, *DT* Deceleration time, *CMR* Cardiovascular magnetic resonance imaging, *RVEF* Right ventricular ejection fraction, *SVI* Stroke volume index, *CI* Cardiac index, *a-vO*_*2*_ *diff* Arterio-venous oxygen difference. ^a^ *n* = 7; ^b^ *n* = 11; ^c^ calculated using peak VO_2_ from cardiopulmonary exercise test and peak cardiac output from exercise CMR

### Cardiac reserve

**Fig. 2:**
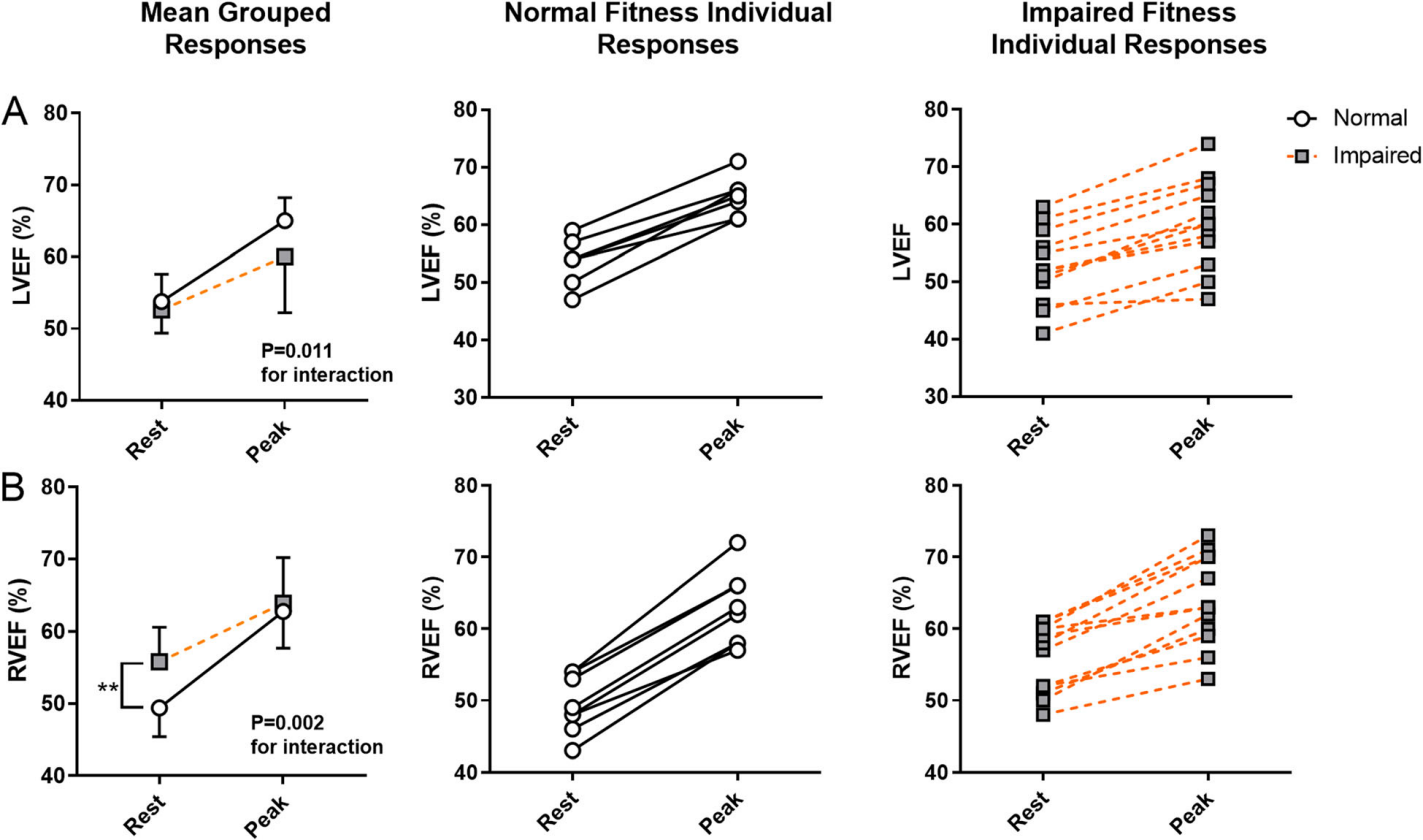
*Individual cardiac contractile response to exercise in pediatric cancer survivors with normal or impaired cardiopulmonary fitness*. Survivors with impaired fitness had a blunted increase in left ventricular ejection fraction (LVEF) from rest to peak exercise (Fig. 2a). In contrast, resting right ventricular ejection fraction (RVEF) was lower in survivors with normal fitness, but showed a greater augmentation from rest to peak exercise (Fig. 2b)

Results related to the central hemodynamic response and contractile reserve are presented in Fig. 1a-c and Fig. 2a-b, respectively. Survivors with impaired fitness had a reduced increase in cardiac index (CI) from rest to peak exercise (Fig. 1a; group-by-exercise response interaction *P* = 0.001), such that CI at peak exercise was 24% lower than survivors with normal fitness (Table 3: 6.8 ± 1.2 vs 8.9 ± 1.6 L/min/m^2^, *P* < 0.01). The impaired CI response appears to be due to differences in SVI reserve (Fig. 1b) as the heart rate response was similar (Fig. 1c). Specifically, survivors with impaired fitness had a markedly attenuated SVI response to exercise (group-by-exercise response interaction *P* = 0.001) and a reduced peak SVI (Table 3: peak exercise SVI 27% lower, *P* < 0.01). Those with normal fitness showed a significantly higher augmentation of both LVEF and RVEF (Fig. 2a and b, group-by-exercise response interaction *P* = 0.011 and *P* = 0.002, respectively). The difference in LVEF response appears to be due to a higher peak LVEF in the normal fitness group (*P* = 0.069) as the resting LVEF was similar (*P* = 0.66). In contrast, the difference in RVEF response is largely due to a lower resting RVEF in the normal fitness group (*P* = 0.006), and a similar RVEF between groups at peak exercise (*P* = 0.68). A video providing an example of the biventricular exercise response assessed by exercise CMR in a survivor with normal fitness and a survivor with impaired fitness is shown in Additional file 1. Those with impaired fitness also had significantly lower a-vO_2_ difference at peak exercise (12.9 ± 2.5 vs 16.3 ± 3.3, *P* = 0.016).

### Associations between exercise capacity with central and peripheral function

Associations between peak VO_2_ and measures of cardiac and peripheral function are displayed in Table 4. There were strong associations between peak VO_2_ and measures of cardiac reserve (peak CI and peak SVI) and peripheral oxygen extraction. In contrast, there were no significant associations between peak VO_2_ and resting echocardiographic and CMR indices of cardiotoxicity (resting LV FS, LVEF and GLS).

**Table 4:** Correlations between peak VO_2_ with cardiac and peripheral measures

Measure	R^**2**^	***P***-value
***Peak exercise measures***		
Peak CI	0.59	< 0.001
Peak SVI	0.67	< 0.001
a-vO_2_ diff	0.71	< 0.001
***Resting measures***		
LVEF	0.01	0.55
LV FS	0.01	0.75
Echocardiographic GLS	−0.10	0.19
CMR GLS	−0.03	0.49

Abbreviations: *CI* Cardiac index, *a-vO*_*2*_ *diff* Arterio-venous oxygen difference, *LVEF* Left ventricular ejection fraction, *LVFS* Left ventricular fractional shortening, *GLS* Global longitudinal strain, *CMR* Cardiovascular magnetic resonance

## Discussion

The main finding from this study was that the classification of pediatric cancer survivors into normal or reduced cardiopulmonary fitness identifies two distinct phenotypes. Specifically, those with normal fitness have a significant augmentation of CI, which occurs due to a significant increase in both heart rate and SVI. In contrast, survivors with impaired fitness have a blunted increase in CI that is primarily driven by increases in heart rate and minimal change in SVI. These differences in fitness and exercise cardiac reserve exist despite no significant differences between groups in measures of cardiac function currently used to quantify heart failure risk and guide clinical decision making.

There is growing awareness of the limitations for the current approach of cardiotoxicity screening in pediatric cancer survivors [6, 8]. Current guidelines are centered on detecting > 10% reductions in resting fractional shortening or LVEF [6, 8], however there is little evidence to show that this approach reliably predicts subsequent cardiovascular morbidity and mortality [6]. Furthermore, reductions in these measures that remain within the normal range show little correlation with exercise capacity or quality of life [23]. We have investigated an alternative approach that focuses on peak VO_2_, a holistic measure of cardiovascular reserve that has been shown to correlate with heart failure incidence [24, 25], prognosis [26, 27] and quality of life [28] among healthy and clinical cohorts. On average, our cohort had a peak VO_2_ that was 19% below predicted values for age, gender and height. This is consistent with a number of other studies documenting pediatric cancer survivors previously treated with anthracyclines and/or chest radiation having a peak VO_2_ that is 8–20% below predicted values or matched control subjects [14, 29, 30]. However, by dividing the spectrum of exercise responses into a binary construct, we have identified a group with marked exercise impairment who have demonstrable abnormalities in cardiac function inducible during exercise stress. The reduction in exercise capacity and cardiac function is likely to be a persistent effect of treatment, as cross-sectional studies have reported that between 30 and 79% of long-term survivors measured > 5 years post-treatment have impaired peak VO_2_ [30–32]. Notably, evidence from large prospective studies in non-cancer populations has shown that incident heart failure risk increased by 16% for each 3.5 mL/kg/min decrement in peak VO_2_ [24, 25]. Given the average peak VO_2_ for the impaired fitness group was 12.1 mL/kg/min below predicted, we would infer this group is at substantially greater risk of development of heart failure symptoms over time than the normal fitness group, who on average, achieved almost 100% of predicted peak VO_2_. This ensues despite no significant differences between our two study groups in resting echocardiographic measures of LV FS and LVEF, nor even in more novel echocardiographic and CMR-derived measures of GLS. This highlights that the current standard of care approach to quantifying cardiac function is insensitive to clinically important differences in peak VO_2_ and heart failure risk.

Given that heart failure is typified by an inability of cardiac function to meet metabolic demand, we hypothesized that assessing cardiac function during periods of increased metabolic demand (such as exercise) should be more sensitive to heart failure risk than the assessment of cardiac function at rest when metabolic demands are low. The inclusion of novel exercise cardiac imaging has allowed us to demonstrate cardiac reserve is significantly reduced in pediatric cancer survivors with reduced peak VO_2_. Specifically, we found that survivors with reduced peak VO_2_ showed a blunted increase in LVEF and minimal augmentation of SVI during exercise, resulting in a blunted increase in exercise CI such that peak exercise CI was 24% lower than survivors with normal fitness. Two cross-sectional reports in pediatric cancer survivors have reported similar results, with survivors demonstrating lower exercise SVI and CI than age- and gender-matched controls [33, 34]. However, our study is the first in pediatric cancer survivors to document this relationship with impaired peak VO_2_. A handful of cross-sectional studies have investigated cardiac impairment in long-term pediatric cancer survivors with normal or reduced peak VO_2_ [29, 30, 35]. However results from these studies have been inconsistent, which may relate to the assessment of cardiac function in the resting state. This is supported by findings from our study, in which there was no significant difference in resting LV FS, LVEF or GLS between our two study groups, nor did these measures correlate with peak VO_2_. The largest of these previous studies [35] demonstrated that impaired peak VO_2_ was associated with reduced resting GLS, but not resting LVEF in 1041 long-term (> 10 years) pediatric cancer survivors, supporting the notion that exercise intolerance is a maker of cardiac dysfunction in cancer survivors. However, results from our study suggest that impairments in fitness and cardiac reserve may precede impairment in GLS. The fact that RVEF was paradoxically lower at rest in fitter survivors is consistent with work in athletic populations in which lower resting RVEF is associated with greater fitness [36]. In the current population, this paradox could create diagnostic confusion and again emphasizes the potential errors that are created by reliance on resting measures and the clarity provided by exercise. Taken together, our results highlight the importance of exercise-based imaging approaches to unmask cardiac dysfunction in cancer survivors.

The relationship between exercise capacity and heart failure risk in cancer survivors is a matter of ongoing investigation. Pandey et al. [37] reported that the greatest long-term risk of heart failure was observed in middle-aged adults with reduced exercise capacity and it is reasonable to speculate that those with markedly reduced exercise capacity as an adolescent would be at even greater risk. Peak VO_2_ is determined by a combination of cardiac (central) and non-cardiac (peripheral) factors, and Houstis et al. [38] have shown 97% of heart failure patients referred for exercise testing had significant limitation in both. Furthermore, it was estimated that complete reversal of cardiac impairment would only improve peak VO_2_ by 7%, but complete improvement in peripheral oxygen extraction would improve peak VO_2_ by 27% [38]. This draws interesting parallels to the current study, where we observed survivors with reduced peak VO_2_ had significant limitation in both cardiac function and peripheral oxygen extraction. Previous studies [39, 40] have documented impairment in peripheral oxygen extraction in breast cancer patients receiving anthracycline- and/or taxane-based chemotherapy, however the existence of peripheral impairment in pediatric cancer survivors is a new and important finding. Given the majority of therapeutic strategies for heart failure prevention in cancer survivors are ‘cardiac focused’, these results highlight the need to investigate adjunctive therapies such as exercise training, that can address peripheral limitations [40]. This also highlights the value of an integrative diagnostic measure such as peak VO_2_, which is able to capture the central and peripheral defects that can contribute to exercise intolerance.

The primary limitation to this study is the modestly sized, heterogenous cohort. Whilst the modest cohort size increases the likelihood of a type I error, the alternative view that the differences noted in this study could represent a large effect size, should not be discounted. Indeed, the findings are not driven by outliers (Figs. 1 and 2), and the ability of our exercise measures to detect significant differences in cardiac function in such a small sample supports the higher sensitivity associated with exercise-based assessment of cardiac function. The small sample size also limits our ability to understand the relationships between host and treatment factors which are associated with reduced exercise capacity. Whilst participants had undergone varying degrees of cancer treatment, this represents the variety of real-world clinical contexts by which cancer survivors present with exertional intolerance. Additionally, the prognostic significance of the relationship between reduced peak VO_2_ and cardiac reserve impairment in pediatric cancer survivors is unclear. Whilst we may speculate by drawing on parallels from other forms of heart failure, our findings require further validation to understand the prospective relationship between reduced peak VO_2_ and clinical endpoints such as heart failure incidence and prognosis.

## Conclusions

Pediatric cancer survivors with impaired peak VO_2_ demonstrate reduced cardiac functional reserve, despite having similar resting cardiac function to survivors with normal peak VO_2_. This suggests that measurement of peak VO_2_ or cardiac functional reserve may provide additional benefit in early identification of survivors at increased risk of cardiac dysfunction and heart failure.

## Supplementary information

**Additional file 1 Figure S1**. *Individual resting global longitudinal strain assessed by cardiac magnetic resonance imaging and speckle tracking echocardiography in pediatric cancer survivors with normal or impaired cardiopulmonary fitness*. Individual values (with overlay of mean and 95% CI) demonstrating that survivors with normal and impaired fitness had comparable resting global longitudinal strain (GLS) assessed by cardiac magnetic resonance imaging (CMR) and speckle-tracking echocardiography (STE).

## Data Availability

The datasets used and/or analysed during the current study are available from the corresponding author on reasonable request.

## Abbreviations

2D: Two-dimensional
a-vO_2_diff: Arterio-venous oxygen difference
BMI: Body mass index
CI: Cardiac index
CMR: Cardiovascular magnetic resonance
CPET: Cardiopulmonary exercise test
ECG: Electrocardiogram
exCMR: Exercise cardiac magnetic resonance imaging
FS: Fractional shortening
GLS: Global longitudinal strain
LV: Left ventricle/left ventricular
LVEF: Left-ventricular ejection fraction
Peak VO_2_: Peak oxygen consumption
RER: Respiratory exchange ratio
RV: Right ventricle/right ventricular
RVEF: Right-ventricular ejection fraction
SVI: Stroke volume index
