# Supplementary figures and images for: Exercise cardiovascular magnetic resonance reveals reduced cardiac reserve in pediatric cancer survivors with impaired cardiopulmonary fitness
Source: J Cardiovasc Magn Reson. 2020 Sep 7;22:64. doi: 10.1186/s12968-020-00658-4 (PMC7487601; doi:10.1186/s12968-020-00658-4)

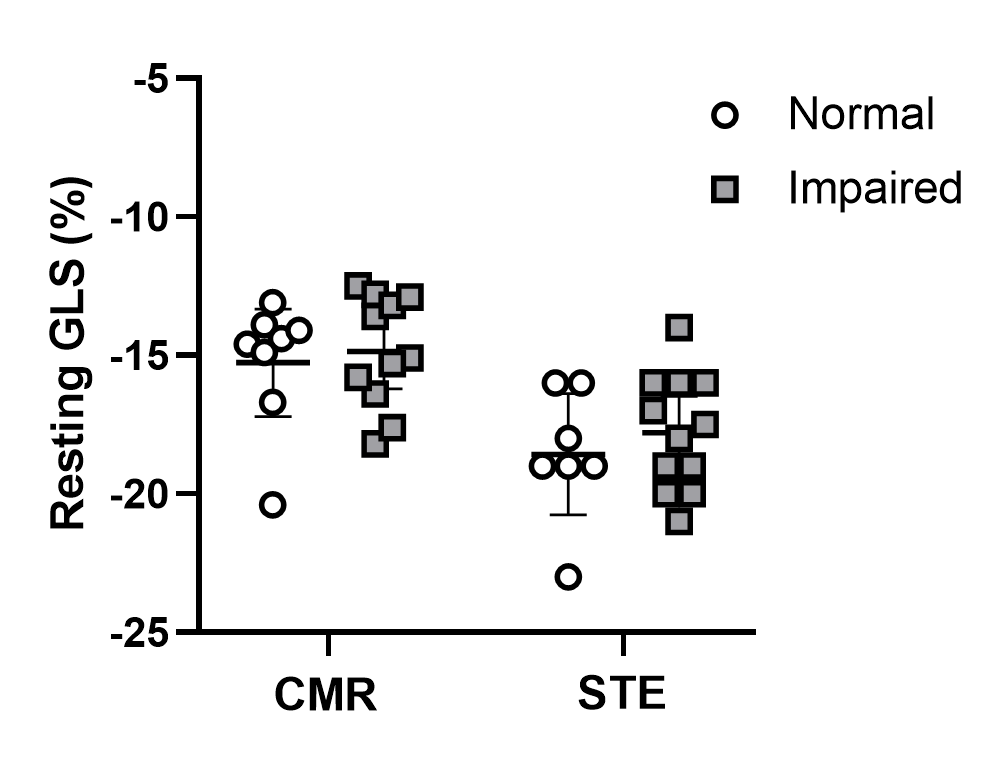

Supplement: Supplementary file 2 — Additional file 1 Figure S1. Individual resting global longitudinal strain assessed by cardiac magnetic resonance imaging and speckle tracking echocardiography in pediatric cancer survivors with normal or impaired cardiopulmonary fitness. Individual values (with overlay of mean and 95% CI) demonstrating that survivors with normal and impaired fitness had comparable resting global longitudinal strain (GLS) assessed by cardiac magnetic resonance imaging (CMR) and speckle-tracking echocardiography (STE). [file 12968_2020_658_MOESM2_ESM.tif]
